# Supplementary material for: Genetic Architecture and Meta-QTL Identification of Yield Traits in Maize (Zea mays L.)
Source: Plants (Basel). 2025 Oct 4;14(19):3067. doi: 10.3390/plants14193067 (PMC12526611; doi:10.3390/plants14193067)
Supplement: Supplementary file 1 [file plants-14-03067-s001.zip › plants-3890154-supplementary.pdf]

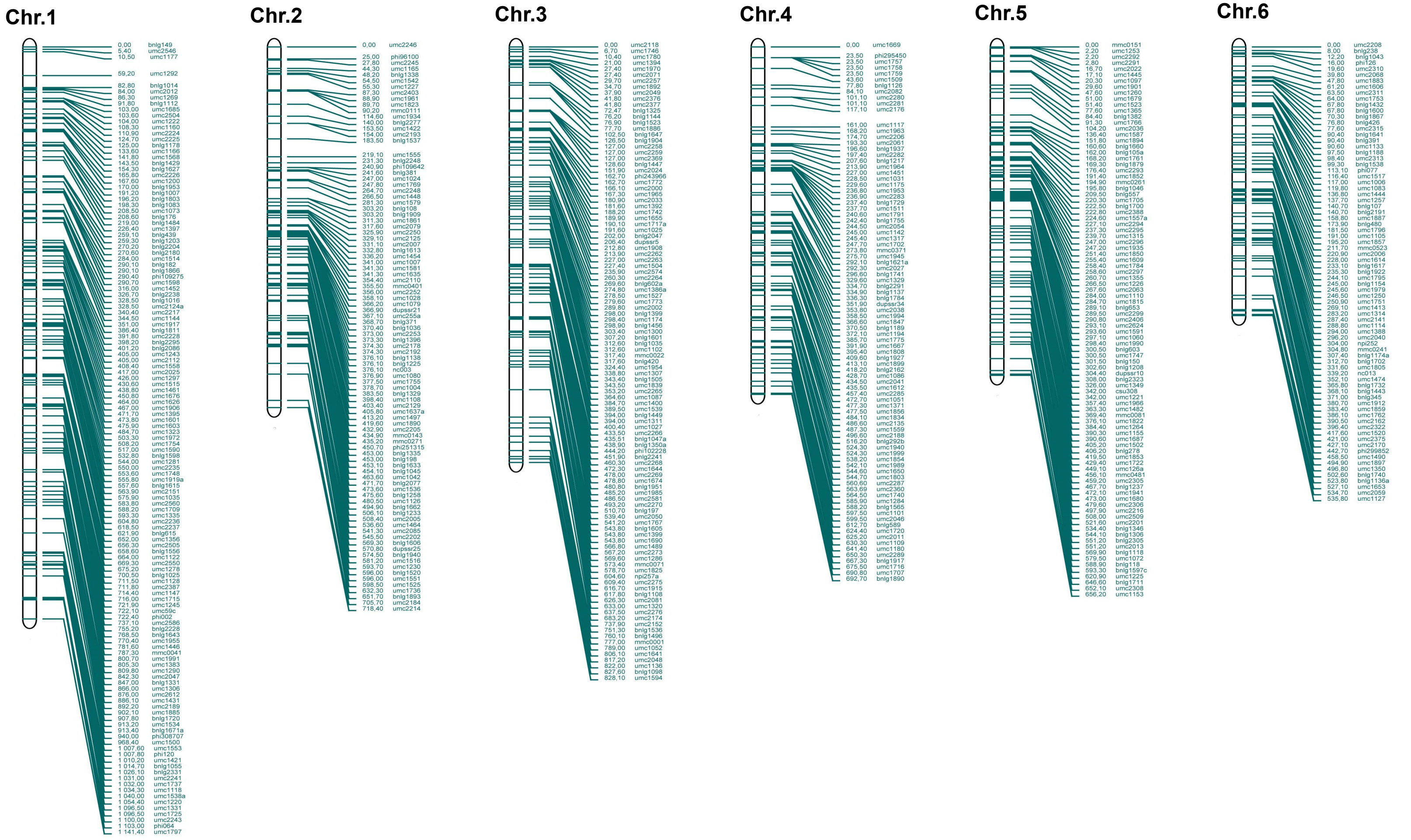

Figure S1. Consensus maps built on fourteen original map datasets. Chr.1-6.

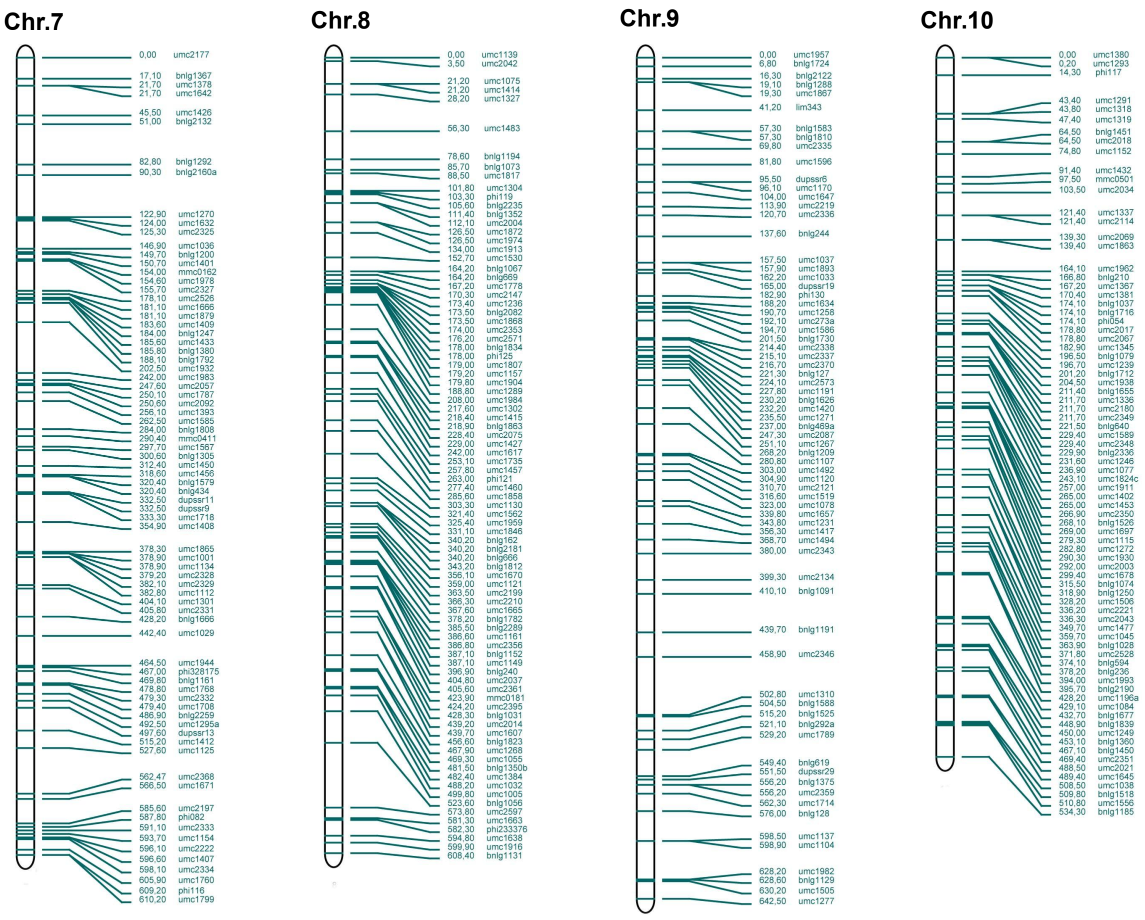

Figure S2. Consensus maps built on fourteen original map datasets. Chr.7-10.

Table S1. The candidate genes related to the regulation of corn yield formation identified within the MQTL interval.

MQTL	Gene_ID	Chr.	Start Site	End Site	Description
MQTL1	*Zm00001d027893*	1	16948608	16955122	Lipoxygenase9
	Zm00001d027904	1	17186476	17190166	3-ketoacyl-CoA synthase
MQTL2	*Zm00001d028325*	1	30793737	30799897	Brassinosteroid synthesis1
	Zm00001d028365	1	32245893	32249856	Cyclin-J18
MQTL3	*Zm00001d029893*	1	92785998	92787072	E3 ubiquitin-protein ligase CCNB1IP1 homolog
	Zm00001d029970	1	97044621	97046748	Cyclin-U4-2
	Zm00001d029983	1	97416254	97419693	Beta-amylase 3 chloroplastic
	Zm00001d029996	1	98331414	98332553	E3 ubiquitin-protein ligase ATL4
	Zm00001d030021	1	100085601	100090561	BR-signaling kinase 2
	Zm00001d030038	1	101381149	101382079	E3 ubiquitin-protein ligase ATL6
MQTL4	*Zm00001d031222*	1	182596026	182597609	Cell Division Protein AAA ATPase family
	Zm00001d031261	1	184229339	184230472	E3 ubiquitin-protein ligase ATL6
	Zm00001d031290	1	185334136	185334576	Probable E3 ubiquitin-protein ligase XERICO
MQTL5	*Zm00001d032046*	1	210548810	210553536	Cytokinin oxidase10
	Zm00001d032078	1	211772301	211796592	Cell division cycle 2-related protein kinase 7
	Zm00001d032088	1	212207299	212207712	SAUR33-auxin-responsive SAUR family member
	Zm00001d032091	1	212248940	212249338	SAUR33-auxin-responsive SAUR family member
	Zm00001d032094	1	212314858	212315259	SAUR33-auxin-responsive SAUR family member
	Zm00001d032117	1	213685223	213690923	Cell division control protein 48 homolog D
	Zm00001d032128	1	213904619	213907090	Cyclin-A1-1
MQTL6	*Zm00001d032576*	1	231120510	231123615	Protochlorophyllide reductase C chloroplastic
	Zm00001d032664	1	233548082	233551762	Cytokinin oxidase6
	Zm00001d032683	1	234266863	234271357	Auxin response factor 16
	Zm00001d032732	1	236508473	236511638	cyclin-dependent kinase inhibitor1
	Zm00001d032734	1	236583915	236593009	No pollen germination related 2
	Zm00001d032776	1	237886808	237891037	Cellulose synthase10
MQTL7	*Zm00001d033580*	1	267761424	267763217	Indol-3-ylacetyl glucosyl transferase1
	Zm00001d033585	1	267905764	267909606	Leaf permease1
	Zm00001d033649	1	269615026	269618990	Beta-glucosidase 44
	Zm00001d033651	1	269673768	269682674	Beta-glucosidase
	Zm00001d033680	1	270918085	270919977	Dwarf plant8
	Zm00001d033749	1	272717186	272719728	Defective kernel35
MQTL8	*Zm00001d034569*	1	296489820	296491595	Cytokinin-O-glucosyltransferase 2
	Zm00001d034629	1	298422859	298427050	Tasselseed6
MQTL9	*Zm00001d002028*	2	4663113	4666196	Protein FATTY ACID EXPORT 2 chloroplastic
MQTL10	*Zm00001d002788*	2	22629133	22631572	Cyclin-U4-2
	Zm00001d002826	2	23922277	23922813	SAUR11-auxin-responsive SAUR family member
	Zm00001d002830	2	24145621	24150188	Beta-fructofuranosidase 1
	Zm00001d002852	2	24694871	24699686	Sucrose synthase 3

	Zm00001d002856	2	24825537	24826490	E3 ubiquitin-protein ligase RMA1
	Zm00001d002929	2	26786830	26791581	Auxin response factor 17
	Zm00001d002970	2	28350907	28355040	Silkless ears1
	Zm00001d002989	2	28930734	28935603	Cytokinin oxidase12
	Zm00001d003011	2	29384400	29385005	Auxin response factor 16
	Zm00001d003018	2	29562998	29563381	SAUR-like auxin-responsive protein family
	Zm00001d003028	2	29759764	29760758	Cyclin-dependent kinase B1-1
MQTL11	*Zm00001d003380*	2	42239268	42240320	Gibberellin-regulated protein 14
	Zm00001d003383	2	42358236	42364264	Auxin-responsive protein [Source:UniProtKB/TrEMBL;Acc:A0A1D6E906]
MQTL12	*Zm00001d005011*	2	153601632	153727710	Embryo defective 1379
	Zm00001d005127	2	159740529	159770203	E3 ubiquitin-protein ligase UPL1
	Zm00001d005154	2	161423330	161427064	Putative E3 ubiquitin-protein ligase RF4
	Zm00001d005293	2	168005838	168007927	Cyclin11
	Zm00001d005367	2	171114902	171115709	Cell division control protein 48 homolog E
	Zm00001d005369	2	171122914	171123806	E3 ubiquitin-protein ligase RGLG1
	Zm00001d005387	2	171976326	171978043	Growth-regulating factor 6
	Zm00001d005393	2	172153302	172161498	E3 ubiquitin-protein ligase
	Zm00001d005487	2	175631316	175632091	Cyclin-related
	Zm00001d005551	2	178279826	178280397	E3 ubiquitin-protein ligase ORTHRUS 2
	Zm00001d005562	2	178526040	178526833	Cell division cycle 20.1 cofactor of APC complex
	Zm00001d005580	2	179433183	179436311	Putative E3 ubiquitin-protein ligase RING1a
	Zm00001d005594	2	180121139	180123254	Sugar transport protein 14
MQTL13	*Zm00001d005853*	2	190929451	190931113	Gibberellin receptor GID1
	Zm00001d005890	2	191852046	191853897	Alpha-amylase 1
	Zm00001d005936	2	193409296	193410477	E3 ubiquitin-protein ligase ATL6
	Zm00001d006011	2	195231015	195246454	E3 ubiquitin-protein ligase UPL5
MQTL14	*Zm00001d007060*	2	221010079	221015469	E3 ubiquitin-protein ligase SP1
	Zm00001d007078	2	221769030	221780225	Sugar transport protein 1
	Zm00001d007100	2	222255509	222256819	Small kernel1
MQTL15	*Zm00001d039318*	3	1628530	1630150	Probable membrane-associated kinase regulator 2
MQTL16	*Zm00001d039717*	3	12897204	12900576	Nucleotide/sugar transporter family protein
	Zm00001d039777	3	14518943	14519897	Cell number regulator 8
	Zm00001d039791	3	14788097	14788336	Auxin responsive protein
MQTL17	*Zm00001d040044*	3	24406987	24407382	E3 ubiquitin-protein ligase [Source:UniProtKB/TrEMBL;Acc:A0A1D6MME5]
	Zm00001d040046	3	24643431	24647894	E3 ubiquitin-protein ligase SINA-like 10
MQTL18	*Zm00001d041049*	3	92907263	92913722	E3 ubiquitin-protein ligase SINA-like 10
	Zm00001d041056	3	93516867	93523481	Auxin response factor 2
	Zm00001d041067	3	94632377	94634331	sugars will eventually be exported transporter13c
	Zm00001d041073	3	95280130	95310416	E3 ubiquitin-protein ligase RGLG1
	Zm00001d041105	3	97492098	97509622	E3 ubiquitin-protein ligase UPL1
	Zm00001d041244	3	107893262	107893994	E3 ubiquitin-protein ligase RGLG1
	Zm00001d041345	3	113725965	113728247	Cyclin-D5-1
	Zm00001d041416	3	118101071	118105468	Auxin-responsive protein IAA14

	Zm00001d041418	3	118360550	118362225	Auxin-responsive protein IAA4
	Zm00001d041462	3	121836564	121836986	SAUR-like auxin-responsive protein family
MQTL19	*Zm00001d043164*	3	190397962	190400788	Cyclin-B1-4
	Zm00001d043183	3	190815534	190816511	Probable E3 ubiquitin-protein ligase RHC1A
	Zm00001d043206	3	191697538	191718032	Putative E3 ubiquitin-protein ligase RING1a
MQTL20	*Zm00001d043795*	3	210370495	210371606	Glutathione S-transferase GSTU6
	Zm00001d043800	3	210461398	210469715	LEAF RUST 10 DISEASE-RESISTANCE LOCUS RECEPTOR-LIKE PROTEIN KINASE-like 1.1
MQTL21	*Zm00001d049001*	4	12228923	12230158	Protein FATTY ACID EXPORT 1 chloroplastic
MQTL22	*Zm00001d050303*	4	80186398	80188770	Cell division control protein 48 homolog D
	Zm00001d050344	4	82553255	82555347	Callose synthase 7
	Zm00001d050371	4	84598981	84600903	Cytokinin hydroxylase
	Zm00001d050475	4	91278970	91282052	Cyclin-D2-1
	Zm00001d050483	4	91998934	92018898	Auxin-independent growth promoter
	Zm00001d050507	4	94918988	94919425	SAUR36-auxin-responsive SAUR family member
	Zm00001d050577	4	101826608	101828504	sugars will eventually be exported transporter15a
	Zm00001d050768	4	121766673	121769040	Two-component response regulator ARR16
	Zm00001d050781	4	122461155	122466889	Auxin response factor 1
	Zm00001d050860	4	127232372	127244717	Sugar transport protein 5
	Zm00001d050873	4	127915099	127918352	Sucrose transporter GRMZM2G106741
	Zm00001d050899	4	129065452	129066520	Cell number regulator 2
	Zm00001d050995	4	135919974	135922107	Cell division control protein 48 homolog D
MQTL23	*Zm00001d052422*	4	189656344	189657108	Pollen allergen Cyn d 23 [Source:UniProtKB/TrEMBL;Acc:B4FBU1]
MQTL24	*Zm00001d053436*	4	231344745	231345618	Cytokinin dehydrogenase 6
MQTL25	*Zm00001d053617*	4	236577171	236580231	Cytochrome P450 734A1
MQTL27	*Zm00001d015905*	5	130027454	130030346	Sugars will eventually be exported transporter4a
	Zm00001d015912	5	130787030	130789864	Sugars will eventually be exported transporter4c
	Zm00001d015914	5	130947371	130950710	Sugars will eventually be exported transporter4b
	Zm00001d015952	5	133821714	133825615	E3 ubiquitin-protein ligase SINAT4
MQTL28	*Zm00001d016838*	5	177880117	177886035	Auxin response factor 1
	Zm00001d016895	5	179608070	179609074	Gibberellin receptor GID1L2
	Zm00001d016901	5	179641467	179642510	E3 ubiquitin-protein ligase ATL6
	Zm00001d016919	5	180058257	180059691	Sugar transport protein 5
	Zm00001d016938	5	180472917	180476181	sucrose transporter GRMZM2G081589
MQTL29	*Zm00001d017725*	5	205294819	205296713	Cell division cycle 20.1 cofactor of APC complex
	Zm00001d017742	5	205734245	205737675	Growth-regulating factor 6
	Zm00001d017743	5	205749775	205753794	Cell division control protein 48 homolog D
MQTL30	*Zm00001d035465*	6	28058154	28062105	E3 ubiquitin-protein ligase RHF2A
MQTL31	*Zm00001d037979*	6	144060572	144071744	Anaphase-promoting complex subunit 1
	Zm00001d037981	6	144086688	144129140	Anaphase-promoting complex subunit 1
MQTL32	*Zm00001d038358*	6	155277368	155285524	Delta-1-pyrroline-5-carboxylate synthase B
MQTL33	*Zm00001d019230*	7	23054461	23065589	Sister of indeterminate spikelet1
	Zm00001d019317	7	27567011	27572466	Cellulose synthase-8
	Zm00001d019322	7	27771073	27773506	Storage protein

	Zm00001d019342	7	28745288	28767439	E3 ubiquitin-protein ligase ORTHRUS 2
	Zm00001d019479	7	36171251	36178389	Granule-bound starch synthase1b
	Zm00001d019507	7	38301954	38308175	Cellulose synthase-7
MQTL34	*Zm00001d020350*	7	108450013	108451003	Alpha-amylase 1
	Zm00001d020351	7	108484538	108486653	Alpha-amylase 1
	Zm00001d020418	7	112714260	112717015	Cytokinin hydroxylase
	Zm00001d020437	7	114149831	114152030	[Fructose-bisphosphate aldolase]-lysine N- methyltransferase chloroplastic
MQTL35	*Zm00001d021404*	7	151347708	151355546	Putative E3 ubiquitin-protein ligase RF4
	Zm00001d021454	7	152609402	152609851	SAUR55-auxin-responsive SAUR family member
	Zm00001d021455	7	152642179	152642637	SAUR52-auxin-responsive SAUR family member
	Zm00001d021456	7	152644791	152645267	SAUR55-auxin-responsive SAUR family member
	Zm00001d021457	7	152649238	152649669	Auxin-responsive protein SAUR61
	Zm00001d021459	7	152719949	152720386	Auxin-responsive protein SAUR61
MQTL36	*Zm00001d021775*	7	162845891	162848484	Sugar carrier protein C
MQTL37	*Zm00001d009943*	8	91379472	91379927	Late embryogeneis abundant protein%3B Late embryogenesis abundant protein
MQTL38	*Zm00001d010775*	8	127477547	127477795	Auxin-responsive protein SAUR71
	Zm00001d010776	8	127492890	127493392	Auxin-responsive protein SAUR71
	Zm00001d010801	8	128221197	128224007	Sucrose synthase 3
	Zm00001d010815	8	128686829	128689613	Flowering locus K homology domain
	Zm00001d010821	8	128734662	128743577	Starch synthase4
MQTL39	*Zm00001d045776*	9	38478283	38478674	Callose synthase 10
	Zm00001d045887	9	45732153	45749402	E3 ubiquitin-protein ligase makorin
	Zm00001d045888	9	45945932	45946440	Pollen-specific arabinogalacta protein BAN102
	Zm00001d045901	9	46519763	46520276	Sugar transporter ERD6-like 6
MQTL40	*Zm00001d047253*	9	124178172	124184872	Sucrose synthase1
	Zm00001d047269	9	124897628	124898960	Protein EARLY FLOWERING 4
	Zm00001d047331	9	126751541	126752764	late embryogenesis abundant protein-related / LEA protein-related
MQTL41	*Zm00001d047946*	9	145935758	145958162	Cell cycle checkpoint protein RAD17
MQTL42	*Zm00001d023450*	10	5902065	5906458	Cationic amino acid transporter 6 chloroplastic
MQTL43	*Zm00001d025268*	10	111867459	111872204	Protein AUXIN SIGNALING F-BOX 3
	Zm00001d025287	10	112727580	112728092	Pollen-specific protein C13
	Zm00001d025300	10	113290809	113298776	Sucrose nonfermenting 4-like protein
MQTL44	*Zm00001d026402*	10	145302797	145311603	White seedling2
	Zm00001d026415	10	145395974	145401877	Beta-D-xylosidase 4
